# Some Practical Points in the Prevention of Pulmonary Tuberculosis

**Published:** 1911-09

**Authors:** William Lee Secor

**Affiliations:** Recently head of the Department of Physiology and Professor of Therapeutics in the Chicago College of Medicine and Surgery. Now physician in charge of the Kerrville Sanitarium and Hospital, Kerrville, Texas


For Texas Medical Journal.

### Some Practical Points in the Prevention of Pulmonary Tuberculosis.

BY WILLIAM LEE SECOR, PH. D., M. D.,

Recently head of the Department of Physiology and Professor of Therapeutics in the Chicago College of Medicine and Surgery. Now physician in charge of the Kerrville Sanitarium and Hospital, Kerrville, Texas.

The time-worn saying, "an ounce of prevention is worth a pound of cure," is most certainly true, when we are considering the worlds greatest plague—tuberculosis.

Ever since Dr. Koch showed so clearly the relation of the tubercle bacillus to tuberculosis the medical profession has been exerting itself to find a cure for this dread disease. Not only have physicians been laboring faithfully in laboratory and clinic, to this end, but many of the laity have enlisted in the fight and have given most liberally of time and means to further the cause and finally the various States have joined the battle and have, by establishing sanatoria, distributing literature and in various other ways, given material aid to the forces already at work.

While very much is being done in the way of cure and new methods are continually increasing our percentage of recoveries, *we must avoid the very fatal mistake of disregarding prevention.*

If cure is important to the individual and the Commonwealth, prevention is much more important. As I have visited various cities and seen in many the apparent disregard for prevention of the spread of disease, it has seemed to me that the medical profession has a great responsibility in this respect.

Every physician should be a shining beacon light in his community—teaching those who are well how to keep well and training the sick to so care for themselves that they will not menace all who may come near them.

We should not leave problems of sanitation to be solved by the physicians of New York or Chicago or some other large city, for every community, no matter how small, has its own problems that should be solved right on the ground.

The scope of the modern physician reaches far beyond the prescription pad and pencil. His greatest service to his community lies in preventing disease and its consequences. While it may not be very remunerative in dollars and cents, the degree of satisfaction that goes with the knowledge of a duty well performed is the best kind of pay.

Diseases like tuberculosis, typhoid fever, malaria, etc., are preventable, and it is now considered a disgrace to any city and its physicians to have these diseases constantly developing within its borders.

Too frequently physicians take it for granted that patients know how to properly care for themselves and neglect to give their tubercular patients detailed instruction as to the methods of transmission of the disease and directions for preventing its spread.

It is now generally conceded that the most common method of transmission from one individual to another is by means of the secretions of the air passages. It is not necessary for a large amount of sputum or an amount even large enough for ordinary detection to come in contact with the air passages of a susceptible person to endanger them to infection for the invisible moisture of the "dry" or *"unproductive cough"* has been shown to contain bacilli.

Every tubercular patient should provide himself with suitable means for protecting others from the products of his cough and for the disposal of his sputum. While about the house, a metal spit cup of ample proportions should be provided. It should have a tight-fitting spring cover to exclude all flies and should contain an ounce of 5 per cent phenol or formaldehyde solution. These specially constructed cups are far better than a cuspidor, as they may be held near the mouth, insuring the proper deposit of all particles of sputum in the antiseptic fluid and not on the sides of the vessel, where it may be reached by flies or dry up and be wafted in the dust of the air.

The proper disposal of the sputum when away from home and on the street is a very different problem, but even more important, as a large number may be endangered by promiscuous spitting on the streets or even in the gutters.

While I do not believe that, as a usual thing, legislation is as good a method of reform as education, it is quite evident that frequently they must go hand in hand. Spitting on the streets should be punished by a heavy fine in every city and town, as it is now in some of the best regulated ones. Why should we emperil our health, the health of our children and the health of the community at large by allowing a few to contaminate our highways with deadly disease-breeding germs?

While it is true that a tubercle bacillus will be killed by direct exposure to the sun's rays for half an hour or so, and this fact is probably the salvation of many, especially those living in towns that are resorted to in numbers by those afflicted with this malady, yet it is a fact that the bacilli may remain active enough to produce a culture for a long time. Very frequently when a mass of sputum is ejected it contains small lumps or particles of tissue that can not be readily penetrated by the sun's rays and which when placed under the microscope prove to be literally loaded with the tubercle bacilli. These particles dry up and are wafted about in the dust or may be swept up on the long skirts of some lady passing by and carried home to the innocent baby just learning to creep about the floor.

Furthermore, this disease-laden sputum is very frequently deposited in places where the sun's rays do not readily penetrate, as in the gutters among refuse, in fence corners, etc. So that the only safe way is to prevent the spitting, if possible.

When away from home, every tubercular patient should provide himself with small squares of heavy tissue paper or muslin. A fresh square should be used at each expectoration and to shield the mouth at each cough. After the sputum has been received in it, it should be deposited in an oiled silk pocket, which is made to fit the side coat pocket where it is pinned in place by safety pins. Ladies may carry this pocket in the handbag. If no water-proof pocket of this kind is used and the paper or linen squares deposited in the ordinary pocket the clothing will soon become contaminated and be a disease spreader. This water-proof pocket should be disinfected frequently by a 5 per cent phenol or formaldehyde solution, and all paper or muslin squares should be burned.

It is doubtless true that those who are in perfect health can resist infection by the tubercle bacillus unless it enters in overwhelming numbers. But who can say when we are in *perfect health?* When these germs are present it is only necessary for a cold or la grippe to so lower the vitality of the patient that the tuberculosis germs may get a chance to grow. Then in every community there are those who have a low power of resistance, and some who by heredity are predisposed to this disease.

Those of low vital resistance and those who are predisposed should follow a strict regimen that may lead them up to the plain of normal resistance. Every harmful practice of modern business or social life should be given up—late hours, irregular, hasty meals, low-neck, short-sleeved dresses and toe slippers, at unseasonable times, etc., should be given up. Regular hours, plain, nutritious meals and rational clothing are essential. They should sleep with windows wide open, but not in a draft and should keep properly protected from dampness. A daily morning sponge bath of ten seconds to two minutes' duration is beneficial; it should be followed by brisk friction. Exercise enough each day should be indulged in to tire but not exhaust.

I am satisfied that if the foregoing suggestions in prevention were carried out to the letter in every community this widespread malady would be checked, to a considerable extent at least. If the individual would do his full share in aiding the cure and prevention of disease there are many diseases that could be blotted out. But for various reasons the individual will not do his share, so that to a large degree the burden of prevention lies upon the municipality or the community—each town and locality must meet its own problems in its own way.

In Texas three of our most prevalent serious diseases are preventable; they are tuberculosis, typhoid fever and malaria. We know the source of each and how to prevent each, yet in many sections they are just about as common as ever.

In order to eradicate or at least limit the spread of these diseases two things at least seem to me to be essential. First, *efficient sanitation;* not dead ordinances but strictly enforced sane ordinances regulating the pollution of water supply, disposal of sewage, cleaning of stables, draining of stagnant pools, spitting on the streets, etc., and, second, the eradication of the three great disease spreaders—dust, flies and mosquitoes. And to my mind by far the best way to do this is by the liberal use of *oil.* Oil on the streets will prevent all dust and make them not only sanitary but a pleasure to ride on. Oil on stagnant pools will soon do away with the mosquitoes and oil on garbage, manure piles and other fly-breeders will limit to a large extent the house fly. Oil properly applied will change a dusty, dirty, disease-ridden community into a most desirable place of residence. It will pay the citizens big returns upon the amount spent and be a joy forever to be free from dust, fly and mosquito.

